# Airborne Infectious Agents and Other Pollutants in Automobiles for Domestic Use: Potential Health Impacts and Approaches to Risk Mitigation

**DOI:** 10.1155/2016/1548326

**Published:** 2016-11-30

**Authors:** Syed A. Sattar, Kathryn E. Wright, Bahram Zargar, Joseph R. Rubino, M. Khalid Ijaz

**Affiliations:** ^1^Department of Biochemistry, Microbiology & Immunology, Faculty of Medicine, University of Ottawa, Ottawa, ON, Canada K1H 8M5; ^2^CREM Co, 3403 American Drive, Mississauga, ON, Canada L4V 1T4; ^3^RB, 1 Philips Parkway, Montvale, NJ 07645, USA; ^4^Department of Biology, Medgar Evers College, The City University of New York (CUNY), Brooklyn, NY, USA

## Abstract

The world total of passenger cars is expected to go from the current one billion to >2.5 billion by 2050. Cars for domestic use account for ~74% of the world's yearly production of motorized vehicles. In North America, ~80% of the commuters use their own car with another 5.6% travelling as passengers. With the current life-expectancy of 78.6 years, the average North American spends 4.3 years driving a car! This equates to driving 101 minutes/day with a lifetime driving distance of nearly 1.3 million km inside the confined and often shared space of the car with exposure to a mix of potentially harmful pathogens, allergens, endotoxins, particulates, and volatile organics. Such risks may increase in proportion to the unprecedented upsurge in the numbers of family cars globally. Though new technologies may reduce the levels of air pollution from car exhausts and other sources, they are unlikely to impact our in-car exposure to pathogens. Can commercial in-car air decontamination devices reduce the risk from airborne infections and other pollutants? We lack scientifically rigorous protocols to verify the claims of such devices. Here we discuss the essentials of a customized aerobiology facility and test protocols to assess such devices under field-relevant conditions.

## 1. Introduction

For safe driving, we are justifiably concerned with road conditions, weather, air quality outdoors, seat-belt use, and distracted and drunk drivers as well as car and driver fitness. Should we also worry about the quality of air* within* the car? If yes, what risks does it pose and how serious can they be for our health? These issues have come to the fore in recent years with increasing coverage in scientific [1–3] and popular media (Gerba and Maxwell 2013; http://loveyourcarandtruck.com/wp-content/uploads/2013/09/germs-in-cars.pdf).

In general, the inside of an automobile is a confined and often shared space, and several reports in the past decade indicate that its occupants thus face a higher risk of exposure to a variety of airborne infectious agents [1–3], allergens [4], endotoxins [5], and volatile organic chemicals (VOCs [6]) alone or in various combinations with possible harm to health. This is at a time when the global number of automobiles on the road is at an unprecedented level (International Organization of Motor Vehicle Manufacturers, OICA; 2015; http://www.fourin.com/english/info/oica.html) while ongoing societal changes also are increasing our exposure and vulnerability to infectious agents in general [7].

Cars, trucks, and vans are by far the most common and convenient modes of transportation. In North America, for example, ~80% of the commuters use their private vehicles with another 5.6% riding as passengers. With the life-expectancy of 78.6 years in 2014 (U.S. Population Bureau), the average North American spends 4.3 years driving a car! This is equal to driving 101 minutes/day with a lifetime driving distance of about 1.3 million km (nearly 798,000 miles) (http://blog.tempoplugin.com/2013/7-time-consuming-things-an-average-joe-spends-in-a-lifetime/).

## 2. Risk Factors for Exposure to Various Types of Pollutants in the Family Car

A combination of factors (Table 1) should be considered when assessing the risks from exposure to infectious agents while using domestic cars. The risk of exposure to a given infectious agent is directly related to the length of the commute as well as the number of occupants in the car. The age of the occupants of such cars and their immune status may also vary widely, thus affecting the outcome of exposure to any pathogens therein. More information on this is given in another section below.

The overall proportion of individuals with acquired (e.g., HIV), induced (e.g., organ transplantation and cancer therapy), and natural (aging) immunosuppression continues to increase with the attendant impact on susceptibility to infectious agents in general. Those on medication for a number of common ailments (e.g., arthritis and diabetes) also suffer from depressed immune systems. In the US, for example, at least 3.6% of the general population is believed to be immunosuppressed at any given time (http://thebulletin.org/growing-number-immunocompromised).

Driving by its very nature can be a stressful experience, with it being further exacerbated under conditions of heavy traffic and inclement weather. The possible impact of such stressors on rider susceptibility to infectious agents remains unexplored.

The relative concentrations as well as the variety of fine respirable particles with an aerodynamic diameter of <2.5 *μ*m (PM_2.5_) on the road are likely to be higher than inside homes. Inhalation of such particulates including those from tobacco smoke [8] and their retention in the respiratory system can predispose occupants to many respiratory pathogens. Inhaled PM_2.5_ can penetrate deep into lungs and may release nanoparticulates into the blood stream causing inflammation, oxidative damage, vasoconstriction, and cardiometabolic dysfunction [9]. In-car exposure to such particulates and VOCs may occur simultaneously, potentially leading to an additive negative impact on the health of the occupants.

## 3. Objectives

This review will critically assess the available information on the following: (a) the potential for exposure to airborne pollutants in cars with emphasis on infectious agents and possible health risks from such exposure, (b) ways of mitigating the identified health risks, (c) future of the car in the face of changing technology and lifestyles, and (d) identification of knowledge gaps and research needs.

## 4. Scope

In this review, the terms “car for domestic use” and “the family car” both refer to an automobile comprising no more than eight seats in addition to the driver's seat. Such cars account for nearly 74% of the total annual production of motorized vehicles in the world (http://www.worldometers.info/cars/). Light commercial vehicles, heavy trucks, buses, coaches, and minibuses, which represent the remaining 26%, will not be discussed here. Nor will it include cars used primarily as commercial taxi cabs. The available peer-reviewed literature as well as other sources of relevant information will be examined with focus on information published in the past 15 years. Where available, data on family car use in fast-developing and populous countries such as Brazil, China, and India will be given for contrast with current and future trends in North America.

While the major focus here is on the potential airborne spread of infectious agents inside the family car, other airborne pollutants such as allergens, endotoxins, respirable particulates, and toxic chemicals (VOCs) will be considered in relation to their impact on host susceptibility to infections. Other factors that may enhance the susceptibility of car riders to airborne pollutants will also be discussed briefly.

## 5. Current Production and Sale of the Automobile

According to OICA (2015), the global production and sale of motorized vehicles reached a record level of nearly 90 million units in 2014, a >34% increase since 2005! Both production and sales of cars in Asia and the Middle East now account for 50% of the global figures, with China showing an unprecedented increase of +7% in 2014 alone. Between 2001 and 2011, the number of registered family cars in India jumped from 5.3 million to 15.5 million, an increase of >290% (https://www.quandl.com/data/mospi/num_motor_vhcl_20_1-number-of-motor-vehicles-registered-in-india-taxed-and-tax-exempted)! The data for Brazil, another emerging economy, show that between 2004 and 2008 the number of cars per 1,000 inhabitants went from 171 to 210, an increase of 81.4%.

**Figure 1:**
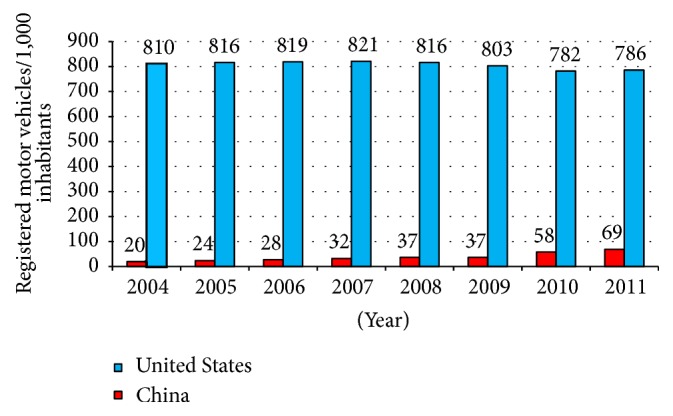
Number of registered motor vehicles/1,000 inhabitants, 2004–2011 (source: World Bank, http://web.archive.org/web/20140806084422/http://data.worldbank.org/indicator/IS.VEH.NVEH.P3?page=1). Note: the term “motor vehicles” here includes cars, buses, and freight carriers.

The world total of passenger cars has now surpassed the billion mark with 174 vehicles/1,000 inhabitants, a >21% increase since 2005 (World Bank 2011; http://data.worldbank.org/indicator/IS.VEH.PCAR.P3). As shown in Figure 1, the number of such vehicles/1,000 in the US has declined from a peak of 821 in 2007 to 786 in 2011; in stark contrast, the number of registered vehicles/1,000 inhabitants in China jumped from 20 to 69 between 2004 and 2011, an increase of 345% (World Bank 2014; http://data.worldbank.org/indicator/IS.VEH.NVEH.P3). In fact, the International Transport Forum (ITF) of the Organization for Economic Cooperation and Development (OECD) predicts that the number of cars and light trucks globally will reach 2.5 billion by the year 2050 (http://www.ipsnews.net/2011/06/bike-vs-car-on-a-hot-planet/).

## 6. Volumes of Passenger and Cargo Compartments of Family Cars

Table 2 presents data on several types and models of family cars and the volumes of their passenger and cargo compartments (https://law.resource.org/pub/us/cfr/ibr/005/sae.j1100.2001.html). The average volume of the passenger compartment inside the family sedan is 115 ft^3^ (3.26 m^3^) while that in the other models is 145 ft^3^ (4.11 m^3^) (https://www.gpo.gov/fdsys/pkg/CFR-1996-title40-vol16/pdf/CFR-1996-title40-vol16-sec600-315.pdf); these values include the space occupied by car seats and other standard features in the passenger compartment. The available volume will also vary depending on the number of riders and the amount of cargo being carried at any given time. The nature and extent of the load a car is carrying will also determine the ongoing air quality along with air movements inside it. These factors, in turn, will directly impact the operation and performance of the car's standard air-handling system as well as that of any air decontamination (“decontamination” is an umbrella term which refers to removal of airborne pollutants by filtration and/or adsorption as well as to inactivation of microbes by chemical (e.g., ozone) or physical (e.g., ultraviolet light) agents) device placed in it. Therefore, these variables must be considered in assessing how well an in-car air decontamination device would perform in concert with its existing air-handling capability under realistic field conditions.

## 7. Sources of Microbes, Their Allergens, and Toxins

**Figure 2:**
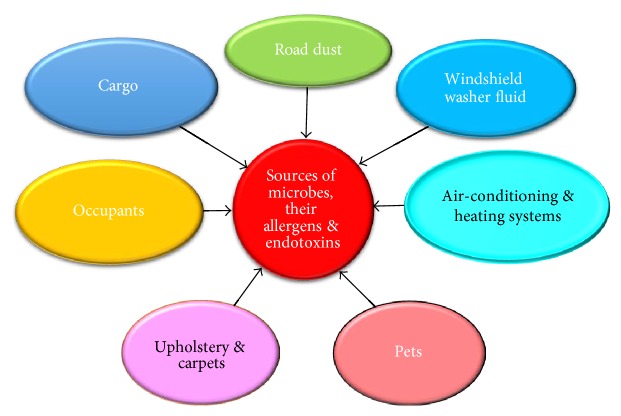
The sources of microbes, allergens, and endotoxins in cars for domestic use.

Figure 2 shows the major sources of microbes, allergens, and endotoxins in the family car. In general, the human occupants are the most common contributors of resident (e.g., staphylococci and propionibacteria) as well as transient (e.g., influenza viruses and rhinoviruses) microbiota. Pets such as dogs may also add to the complement of microbes with potential risks to humans [10].

Dust is by far the most frequent source of environment-based bacteria and fungi along with the allergens and toxins associated with them. Such dust settled on carpets and upholstery may become resuspended, thus contaminating the air and/or other areas within the car. Sufficient levels of moisture from water/food spillage inside the car can also promote the replication of dust-carried microbes. Cargo in the passenger compartment may further contribute to the loading of dust-laden microbes, most of which are unlikely to be directly harmful to humans.

Biofilms formed in car heaters/air conditioners [11, 12] as well as those in windshield washer reservoirs [13] and other areas of the car may release microbes such as legionellae and possibly environmental or nontuberculous mycobacteria (NTM) as well as airborne opportunistic pathogens. Such pathogens may also come from road dust and water in road puddles [14].

Table 3 is a listing of the major types of microbes and their sources along with examples of those that may be found in the family car. The list includes several known and potential human pathogens. Whereas viruses of human and animal origin can only be spread directly from their respective hosts, other pathogens (except* Mycobacterium tuberculosis*) can replicate in various parts of the family car under suitable environmental conditions with biofilms representing a particularly significant niche. Therefore, any successful risk mitigation strategy must include ways of reducing the possibility of microbial growth within the car and also be capable of inactivating those potential pathogens released from biofilms. Regular cleaning and maintenance of the car are also crucial to keep the microbial load inside it as low as possible. On the rare occasion when animals such as chickens and pigs (potential sources of influenza virus, e.g.) are being transported in the family vehicle, extra care would be needed to reduce the risk from exposure to any human pathogens that they may carry.

Many types of NTM, which are common in biofilms [15] and dust [16], are increasingly being recognized as opportunistic human pathogens [17–19]. Surprisingly though, there is virtually no information on their recovery from inside the family car. This may, in part, be due to the extra effort needed to find them in environmental samples. Any future studies on the microbiota in the family car should include a search of NTM, and a suitable surrogate for them should also be added to the list of microbes to test devices for in-car air decontamination.

The microbes listed in Table 3 are only a fraction of those found in the family car detected by culture- [1, 4, 20] and non-culture-based [21] means. However, the health implications of many of them remain unknown. Nonetheless, the inside of a family car is unique in the mélange of airborne pollutants it often contains with possible simultaneous exposure of its occupants to them. Thus, any true assessment of risk must consider the possible additive negative impact of such combined exposures [22].

## 8. Infectious Agents of Concern

As shown in Table 3, several types of known or potential microbial pathogens may be found inside the family car. But we are unaware of any published studies linking cases of any type of infection from exposure to the atmosphere inside the family car. This may well be due to the difficulties of generating such information, especially in view of the likelihood of such exposures resulting in a very limited number of cases. The following, therefore, is a critical look at the* suspected* health impacts of in-car infectious agents.

### 8.1. Legionellae

Legionnaires' disease (LD), caused by an environment-based Gram-negative bacterium, is a serious and potentially fatal lung infection [23, 24]. While several species of the genus* Legionella* can cause the disease,* L. pneumophila* is responsible for >90% of the cases. Pontiac Fever is a milder and generally self-limiting form of lung infection also caused by members of the genus* Legionella* [25]. The bacteria are common in biofilms, which are slimy layers of a mixture of microbes growing on surfaces submerged [26] in water or other liquids [27]. Inhalation of fragments of biofilms containing microbes such as* Legionella* poses health risks particularly to those debilitated due to age, chronic smoking, immunosuppression, or other underlying factors [23]. Though LD can be readily treated with antibiotics, its clinical diagnosis is often difficult. A noteworthy feature of LD is that it can be acquired only after inhalation of the bacteria released from biofilms and that an infected individual cannot pass the infection on to others to give rise to secondary cases [28]. Since their discovery in 1976,* Legionella* spp. are being incriminated in increasing numbers in cases of pneumonia all over the world [29]. As summarized below, they are also emerging as a major concern for airborne infections from automobiles.

The first report on possible links between LD in intercity bus drivers and water-based biofilms in evaporative condensers of air conditioners was published by Polat et al. [30]. Such drivers and their assistants were considered at a higher risk due to their direct and prolonged exposure to the buses' air-conditioning and air-circulating systems. The sera of 19% (12/63) of the drivers were positive for antibodies against* L. pneumophila* with no assistants (0/16) showing seropositivity. Water samples from the air conditioners of the buses with seropositive drivers were all negative for* Legionella* spp. by culture and by the polymerase chain reaction (PCR). Although this study regards legionellosis as an occupational risk factor for intercity bus drivers, its findings are just too preliminary to justify that conclusion, especially with no evidence for the presence of the etiological agent(s) in the water samples. Also, no information is given on a cohort engaged in other occupations for comparison.

The condensate from a malfunctioning car air conditioner is believed to have been the source of L.* pneumophila* in one case [31]. Other professional drivers appear to be at an increased risk of LD [32]; nearly 33% of cabin air filters from various types of cars they tested were colonized with* L. pneumophila*, the major etiological agent of the disease, suggesting such filters as hitherto unrecognized reservoirs for the pathogen.

A molecular analysis of swab samples from the evaporator compartments of the air-conditioning system of scrapped cars found 50% (11/22) of them to be positive for* Legionella* [33]. They also tested healthy subjects who were mainly employees of regional transportation companies for antibody to* L. pneumophila*; the participants also completed a questionnaire. The prevalence of microplate agglutination titres of 1 : 32 was significantly higher in the employees who sometimes used car air-conditioning systems. Although their findings did not prove a direct link between* Legionella* spp. in the car evaporators and LD, the findings point to a potential risk of LD in car air-conditioning systems.

Bacteria released from biofilms in windshield washer reservoirs may include legionellae [13, 34, 35], which may enter cars from road dust and water in road puddles as well [14, 33].

Though certain of the studies summarized above allude to risk of LD for professional drivers while the others have found components of an automobile's liquid and air-handling systems positive for legionellae, the relevance of their findings to air quality in the family car remains to be established.

### 8.2. Other Types of Bacteria and Fungi

An investigation on malodors associated with air-conditioning systems in automobiles found the heat exchanger fins of 45 evaporators from seven different regions of the world to be coated with biofilms [11]. The biofilms were analyzed and found to contain a wide variety of bacteria including potential human pathogens such as members of the genera Sphingomonadales, Burkholderiales, Bacillales, and* Stenotrophomonas*. Quite remarkably, no* Legionella* were detected. While the tested samples may indeed be negative for the bacteria, other possible reasons for the failure to detect them may be the presence of inhibitory chemicals and sequestration of the bacteria in associated fungi [36].

Li et al. [37] note the lack of data on risks associated with the exposure to microbial aerosols from automobile air conditioners (AC). They collected samples of dust from AC and engine filters from 30 automobiles in four coastal locations in China and analyzed them for bacteria, fungi, and endotoxins. Irrespective of the location of the tested vehicles, the dust from their AC filters revealed relatively high levels of bacteria (~26,150 CFU/mg), fungi (~1,287 CFU/mg), and endotoxins (~5527 EU/mg). More than 400 types of bacterial species were detected including opportunistic pathogens, such as* Acinetobacter*,* Bacillus*,* Pseudomonas*, and* Stenotrophomonas*. Some 18 types of allergenic fungal species were also found in abundance.

The coastal nature of the study's locations (Beijing, Guangzhou, Haiku, and Shanghai), with their typically high levels of relative humidity (RH), may have influenced the moisture levels on the filters, thus favoring microbial survival and growth on them. The levels of endotoxins normally correspond directly to the concentration of Gram-negative bacteria at a given site, and this is most likely reflected in the abundance of such organisms in the tested samples. It would be worthwhile to conduct such studies in drier locations for comparison. Environmental mycobacteria, emerging opportunistic pathogens of humans, are notable for their absence in this study, possibly because the special culture media/conditions and molecular test methods required for them were not a part of this investigation. In general, however, this investigation is thus far among the few comprehensive ones to assess the microbial loading of air filters in automobiles. Its findings also show the benefits of air conditioners in reducing the levels of airborne particulates in automobiles.

The influence of AC and heating systems on the levels of airborne bacteria and fungi inside automobiles has been assessed [1]. Soon after the start of the AC systems, there was an increase in the levels of airborne microbes due to the purging of their pipes and also as a result of the resuspension of accumulated dust inside the cars. This was followed by significant drops in the aerosol levels in the next 5–35 minutes. In contrast, the heating systems did not show the initial increase in microbial aerosols, possibly because of microbial inactivation by the heating coils. The data in this study are based on five cars and the collection of 2-minute air samples using a single-stage Andersen sampler. Such a sampler is much less appropriate than a slit-to-agar (STA) air sampler designed to show a time-related distribution of airborne particles. Nevertheless, they detected several species of airborne fungi with* Alternaria*,* Aspergillus*,* Cladosporium*, and* Penicillium* being the most common. The report does not give any details on the types of bacteria or viruses recovered from the air inside the cars.

Microbes growing inside car air conditioners have been found to release VOCs with noxious odors [38], and reductions in moisture levels together with the use of materials refractory to microbial growth have been suggested to remediate this problem.

Though investigations of the microbial content of automobile interiors using culture-dependent and culture-independent (molecular) methods found wide variations in the numbers and types of bacteria among the cars and sites tested,* Staphylococcus* and* Propionibacterium* were the most common and dominant of the over 36 bacterial genera found at the locations sampled [3].* S. aureus* was among the staphylococci isolated with 23% of its strains being resistant to methicillin (MRSA). Coating the steering wheel with a silver-based compound was found to eliminate the presence of culturable pathogenic bacteria. While the use of antimicrobial coatings is a promising way to reduce the risks from microbial pathogens, such an approach currently has several limitations to consider before its wider application [39]; important among these are (a) a limited microbicidal spectrum, (b) potential to generate microbicide resistance, and (c) reduced microbicidal activity in the presence of organic and inorganic matter.

Vonberg et al. [40] examined the influence of AC systems on the microbial quality of air inside automobiles. Even though air-conditioning is a standard feature in many automobiles these days, its impact on the general quality of the air inside requires further exploration. In this 30-month study the influence of fresh and recycled air modes on the content of airborne microbes and mold spores is measured by impaction in a high flow air sampler; a laser counter recorded the number of particles (0.5–5.0 *μ*m diameter). Each sampling was for 1 minute only with the collection of 50 L of air. The microbial content of the outside air was always higher than that inside. Soon after the start of the AC system, the levels of microbes, mold spores, and the particulates registered reductions of 82%, 83%, and 88%, respectively. Remarkably, operating the AC with fresh or recirculated air showed no significant difference in air quality, possibly due to the action of the air filter. This study underscores the need for regular maintenance of the system and replacement of air filters in it for optimal benefit.

The concentration of airborne fungi inside automobiles was tested under the following four conditions [20]: (1) window closed without AC and circulation, (2) window open without AC and circulation, (3) windows closed with only circulation on, and (4) windows closed with only AC on. Under the last condition, the mean respirable fraction was 83.3%, with a median diameter of the fungi being 1.73 *μ*m. The authors suggest that more attention be paid to these smaller fungi which can readily enter the alveoli and probably lead to allergic alveolitis.

Gerba and Maxwell (http://loveyourcarandtruck.com/wp-content/uploads/2013/09/germs-in-cars.pdf) swabbed 11 different types of surfaces in 100 cars from four different states (Arizona, California, Florida, and Illinois) and the Washington, DC, area of the US for bacteria and fungi. The findings, including hitherto unreported aspects of microbes in cars, are summarized in Table 4. Overall, the numbers of aerobic bacteria isolated ranged from <10 to 8.0 × 10^5^ colony-forming units (CFU)/4 inches^2^ (25.8 cm^2^). Though the types of bacteria isolated and the relative frequency of their isolations are not given, MRSA is stated to have been recovered from 2% of the automobiles. The fungi isolated belonged to 10 different genera and, of the total fungal isolates,* Aspergillus* species represented 64% (37/58).

Though the study by Gerba and Maxwell (http://loveyourcarandtruck.com/wp-content/uploads/2013/09/germs-in-cars.pdf) focused entirely on surfaces, their findings have implications for the quality of in-car air as there is frequent interchange of microbial contamination on environmental surfaces and air. In view of this, the overall impact of any in-car air decontamination technology would be greater if it can be shown to reduce surface contamination as well. In fact, we observed a reduction in experimentally aerosolized bacteria in a room-size chamber when an air decontamination device was operational [41]. The above-mentioned findings of Gerba and Maxwell, though as yet unpublished in peer-reviewed literature, are also to be regarded as a general indicator of the levels and types of microbial contamination in the family car with no assumption of any associated health risks.

### 8.3. Influenza Viruses

Influenza viruses possess a lipid-containing envelope making them relatively fragile, unstable in the environment, and also susceptible to the action of even mild detergents [42]. In spite of the long history of influenza and the well-known ability of influenza viruses to cause frequent epidemics and pandemics, the precise means of spread of these viruses in nature as well as the relative importance of various types of vehicles in their transmission still remain unclear [43]. Experimental [44] and epidemiological [43, 45] studies strongly support the airborne spread of influenza viruses; while fomites and hands are also believed to play a role in their spread, the evidence for the airborne spread of influenza requires strengthening.

The report by Knibbs et al. [46] is the only published one dealing with influenza viruses and their possible airborne spread inside cars. They modelled virus spread in view of a suspected case of influenza spread during car travel in Australia [47]. They noted wide variations in the efficiency of air circulation depending on the age and make of the car. Also, the estimated risk of influenza spread ranged from 59% to 99.9% for a 90 min trip when air was recirculated. These findings have implications for the design and operation of any in-car air decontamination device to deal with airborne viruses including the enveloped ones.

## 9. Endotoxins and Allergens

Endotoxins are lipopolysaccharide found in the cell walls of pathogenic (e.g.,* Salmonella* and* Pseudomonas*) and nonpathogenic (e.g.,* Escherichia coli*) Gram-negative bacteria. They can be shed in trace amounts from living cells or released in larger quantities when such cells disintegrate. Injection or inhalation of endotoxins can cause fever, chills, and shock [48].

Wu et al. [49] tested dust samples from the passenger seats of 40 cars as sources of bacterial endotoxins and fungal *β*-(1, 3)-glucan as exposure to such substances could induce respiratory symptoms. Both types of substances were found in each sample at levels potentially unsafe for asthmatics.

It would not at all be unusual to find certain levels of bacterial endotoxins and fungal *β*-(1, 3)-glucan as well as allergens of microbial and nonmicrobial origin inside virtually every family car considering its normal use patterns. What may vary though are the potential negative health impacts of such substances on the rider(s). Any in-car air decontamination device should thus include, in addition to microbial pathogen- and VOC-removal, the ability to effectively reduce the levels of such toxins and allergens for a wider customer appeal.

## 10. Tobacco Smoke and Air Quality

According to the World Health Organization (WHO), between 2007 and 2012 the number of countries imposing restrictions on cigarette smoking increased from 44 to 92 with the population coverage going from 1.045 billion to 2.328 billion (WHO; http://www.who.int/tobacco/global_report/2013/en/). Much progress still remains though considering that there are >200 countries with a total population of well over 7 billion.

The data for 2012 indicated that China is the world's largest overall consumer of cigarettes [50] with >1,700 cigarettes being smoked/person/year; the comparable figure for the US is 1,000. However, the rate in China is expected to go up with the increasing urbanization of the country (Fisher, Washington Post; October 2012).

Cigarette smoke is known to contain over 70 carcinogenic chemicals which can harm not only the smoker but also those exposed to secondhand smoke. Since such “passive smoking” can be particularly harmful to children in the confined space of family cars, the increasing number of jurisdictions in North America and elsewhere has been imposing bans on in-car smoking with children present. With regard to respirable particles, modelling studies show that after smoking one cigarette in a stationary midsize car with the AC off it takes 10 to 60 minutes for the levels to return to their initial values [51]; a part of this reduction is due to adsorption of the particulates to surface and not necessarily due to dilution with fresh air.

In a study in the UK, Semple et al. [52] measured, over a three-day period, levels of fine particulate matter in cars as a marker for secondhand smoke during typical real-life car journeys (lasting 5 to 70 minutes) by 14 smoking and 3 nonsmoking study participants. The use of forced ventilation and opening of car windows were quite common during smoking journeys, but concentrations of respirable particles still exceeded the WHO indoor air quality guidance at some point in the measurement period during all smoking journeys. Children exposed to such levels of fine particulate as a surrogate for secondhand smoke are quite likely to suffer harm to their health reinforcing the need for greater controls on smoking in family cars in particular.

Apart from the increased risk for lung cancer and other health problems [53], exposure to tobacco smoke can exacerbate chronic obstructive pulmonary disease (COPD) [54] and attacks of asthma and also lower the body's resistance to infectious agents such as tuberculosis [8].

## 11. Particulates and Chemicals

In July 2000, the International Center for Technology Assessment (ICTA), based in Washington, DC, published a report based on 23 studies relating to chemical pollutants in the air of passenger compartments of cars (http://www.icta.org/doc/In-car%20pollution%20report.pdf). The ICTA concluded that the levels of several types of airborne chemical pollutants inside the car were higher than those in ambient air. It went on to state that “elevated in-car pollution concentrations particularly endanger children, the elderly, and people with asthma and other respiratory conditions. While it receives little attention, in-car air pollution may pose one of the greatest modern threats to human health.”

Müller et al. [55] note that there continues to be greater emphasis on air pollution from outdoor sources even though many of us spend long periods each day inside homes and in other confined spaces such as the family car. Therefore, they summarized information on exposure to chemicals and particulate matter indoors with emphasis on nonvehicular sources including the impact of tobacco smoke inside automobiles (see the following list). Their review is a relatively recent analysis of the role particulates and other types of chemicals may play in lowering the quality of the air inside cars. 

*Examples of Particulates and Organic Chemicals in the Air Inside Automobiles Which May Either Be Directly Harmful to Health or Lower the Body's Resistance to Airborne Pathogens*

Carbon monoxide
Nitrogen dioxide
Sulphur dioxide
Tobacco smoke
PM_2.5_
Brominated flame retardants
Aliphatic hydrocarbons (methane and propane)
Aromatic hydrocarbons (benzene)
Volatile organic chemicals (formaldehyde, ethanol, and methanol)

Particulate matter, smaller than 2.5 micrometers in diameter, is particularly known as health hazard. Major sources of PM_2.5_ include coal-fired power, steels plants, and car exhaust.

## 12. Mitigating the Risks from Infectious Agents and Other Pollutants inside the Family Car

As should be apparent from the information presented thus far, microbes, chemicals, and respirable particulates from a variety of internal and external sources can impact the atmosphere in the family car with potentially negative consequences on the health of its occupants. So, what possible approaches are there for mitigating such risks?

Table 5 presents a summary of the available approaches with their strengths and limitations. A suitable combination of the approaches listed would be necessary for an optimal positive impact on the health of the car's occupants.

The factors listed in Table 6 must be borne in mind in the development and choice of any device or technology for the decontamination of in-car air. The selection of any such approach must also be based on a thorough premarket assessment using realistic challenges under experimental conditions followed by rigorous field testing. Though it would be highly desirable to show that the use of any such approach also reduces the risk from airborne pollutants in family cars, such studies would be difficult to design and conduct while needing substantial amounts of time and funds to complete successfully.

While many devices are now marketed with claims for in-car air treatment, most are meant for odor removal. Those that claim microbial removal (mostly using HEPA filters with or without an activated charcoal filter) provide virtually no details on how they were tested. This is not surprising considering the absence of any standardized and regulator-recognized test protocol. While the guideline from the US EPA (EPA-HQ-OPPT-2009-0150) relates to indoor air decontamination, it is not directly applicable to assessing in-car air treatment devices. There is, therefore, a need to address this gap by developing robust and scientific valid ways of assessing such devices under field-relevant conditions.

It should also be noted here that space and cost limitations would permit only relatively small devices in family cars in general. However, the potential health benefits of such devices would be greater if they could additionally reduce the levels of airborne allergens, harmful chemicals, and particulates including PM_2.5_.

## 13. Future of the Family Car

For nearly the past eight decades of “modern living” in North America and Europe has made urbanization and car ownership essentially synonymous, owning and driving a car ceased to be luxury long ago and became an everyday necessity for the entire family. However, the negative environmental consequences of the family car's unprecedented popularity have now triggered the “war against the car” with the increasing availability of public transport and rejuvenation of inner cities with high-density dwellings. Though Douglas et al. [56] suggest that increasing concerns with the negative environmental impacts of the private automobile will turn it into the “next tobacco,” this trend is being partly offset by the development and marketing of cars which consume either no or reduced levels of fossil fuel. While these changes may lead to a slow decrease in the level of car ownership in North America and Europe along with reductions in exposure to harmful car exhausts from the burning of fossil fuels, the inside atmosphere of a family car may remain essentially the same with its attendant risks of exposure to infectious agents.

While the overall number of cars in North America and Europe may be declining slowly (Figure 2), it is unlikely that this trend will lead to significant reductions in their numbers anytime soon for the following reasons:

1. The human population is anticipated to reach over nine billion by the year 2050 (United Nations: https://esa.un.org/unpd/wpp/publications/files/key_findings_wpp_2015.pdf), with a corresponding increase in the demand for the family car and the numbers of riders in it. This is clearly indicated by the already skyrocketing numbers of cars in emerging economies such as China and India, as examples.
2. Public transportation continues to be inadequate in the face of growing ridership and still-expanding urban centers.
3. Many decades of investment in building roads to establish and sustain the ever-widening urban sprawl are irreversible. Besides, the ongoing population increases as well as mounting urbanization continue to add to the demand for housing and ancillary infrastructure in areas away from the inner city.
4. For many, the private car still remains the most convenient means of transport for work, shopping, and family outings.
5. The increasing availability and affordability of the “green” family car eliminates much of the “guilt” of owning a car.

## 14. Discussion and Directions for the Future

As summarized in this review, many studies have documented the presence of many types of infectious agents in the air and on surfaces in family cars. Moreover, there continue to be concerns with the human health impacts of respirable particulates including PM_2.5_ and chemical pollutants inside cars and their potential to enhance the susceptibility of humans to infectious agents [22]. However, a crucial gap in our knowledge continues to be the absence of demonstrated links between infectious agents in the air inside cars and any negative impacts on rider health. Such studies, while potentially highly valuable, are generally very expensive and difficult to plan and conduct and yet may not yield unequivocal data. Therefore, any decisions to promote the marketing of in-car air decontamination devices would have to be based on risk assessments considering the quality of the available information. In addition, experimental studies would be needed to generate scientifically valid data on the efficiency and relative merits of available in-car air decontamination technologies under experimental and simulated field conditions to reduce the levels of infectious agents and other airborne pollutants.

Though NTM are common in biofilms, water, dust, and other parts of the environment, there are as yet no reports of their detection in cars. This obvious knowledge gap should be filled by including a search for them in any future studies on the microbiota of family as well as other types of cars.

“Chembioaction” refers to the phenomenon where the combined exposure to a chemical and microbe may result in a more serious health outcome compared to when the host is exposed to either one of them alone [22], which can potentially be exacerbated in immunosuppressed populations. While evidence for it comes from animal experiments and limited epidemiological observations, generating data is inherently difficult. Nevertheless, this fact should be borne in mind in any discussion on the human health impact of in-car air pollution.

Without question, the quality of the air inside a family dwelling is paramount for the health and well-being of its residents. Nonetheless, the air quality in the family car may be subject to certain factors over and above those in a family dwelling. Important among these are the following: (a) a lower ratio of air volume/capita in cars, (b) greater proximity between occupants in cars, (c) more frequent fluctuations in air quality in a moving car based on the terrain, speed, surrounding air quality, and operation of air heating/cooling system, and (d) greater variety and higher quantities of chemical pollutants and respirable particulates along with more frequent and greater fluctuations in RH and air temperature. These differences must be borne in mind when considering the potential benefits of in-car air decontamination technologies.

## 15. Research Needs

The following research needs have come to the fore while reviewing possible health risks from airborne infectious agents in the family car.

First and foremost, our knowledge on the types and levels of airborne infectious agents in the family car remains rudimentary. Further studies with better air sampling technologies are needed to develop a more comprehensive and event-related profile of* viable microbes* under a variety of geographic, traffic, and weather conditions. For example, the deployment of programmable slit-to-agar (STA) air sampling devices [57] would offer the following advantages over liquid impingers and single-stage Andersen air samplers [1]: (1) they can give an event-related distribution of the microbial content by directly and gently collecting the microbial load on the surface of nutrient agar; the agar plate can be incubated for the development of colony-forming units (CFU) without any further manipulations; (2) the sampler can be set to run from a minimum of two minutes to a maximum of five hours depending on the length of air sampling required; (3) any activity resulting in an increase or decrease in the microbial contact in the air is reflected directly on the number of CFU during that period. Such information would be crucial to better assess the airborne exposure of car riders to known or opportunistic (including NTM) bacterial or fungal pathogens. Testing with experimentally generated microbial aerosols will be needed to model the movement of pathogens inside the car under a variety of conditions, including opening of car windows and operation of its air-handling system. Recently published test procedures to assess microbial survival and decontamination in indoor air could be adapted to work with family cars [41, 57, 58].

Laboratory-based testing using simulations of the inside of a typical family car and challenge with experimentally generated aerosols of pathogens or their surrogates would be needed to assess any air decontamination technology under a variety of field-relevant conditions.

Recent studies have reinforced the importance of the microbiome of various settings in understanding the influence of physical and lifestyle changes [59]. The study of the microbiome of the family car under different environmental and use-conditions would be beneficial to assess the impact of different physical/chemical decontamination technologies.

## 16. Conclusions and Recommendations

In general, the inside of a family car is a much more confined space as compared to a typical family dwelling. Cars in general are also under the more direct influence of weather and climate as well as fluctuations in the surrounding atmosphere including health status of the occupants. These factors, along with the makeup and quality of the interior and the activities of its occupants, can impact the chemicals in air as well as in-car air microbiome. The available evidence also suggests that such airborne chemicals and pathogens may work in synergy for greater harm to human health.

Whenever possible, source control must be considered to reduce the levels of pollutants in air along with the installation of any air decontamination technology. While many such devices are already on the market, information on how they were tested to validate their claims remains unavailable in the public domain, thus making it difficult to assess their relative merits and safety features.

Published information on individual cases or outbreaks of adverse health effects from exposure to the air inside cars remains unavailable; this may reflect on the difficulties of designing and conducting investigations to generate such data. However, the available expert opinions and published data on the potential for exposure to pathogens, allergens, and respirable particulates including PM_2.5_ and VOCs inside cars indicate that such risks not only exist but also may increase due to a combination of ongoing societal and environmental changes.

Therefore, consideration should be given to finding suitable means of mitigating such risks through innovative technologies which are not only economical and safe, but also broad-spectrum in their ability to deal with as many types of airborne pollutants as possible. Any such technology will require a thorough assessment in an experimental setting prior to field testing and application. Notwithstanding these factors, the availability and use of the family car are not likely to see any significant reductions any time soon.

## Figures and Tables

**Table 1:** Risk factors for exposure to infectious agents inside family cars.

Factors	Impact
Length of commute	Risk of exposure to harmful airborne contaminants increases in direct proportion to the length of commute
Carpooling	Risk of exposure to harmful airborne contaminants increases in direct proportion to the number of occupants
Immunosuppression	Increasing proportion of the immunosuppressed persons in the general society
Potential hosts	Wide variation in the age & general health status of occupants
Stress of driving	Stress of driving may lower body's general resistance mechanisms
Respirable particulates	Inhalation of such particulates may enhance exposure & susceptibility to infectious agents
Volatile organic chemicals	Exposure to such chemicals may occur simultaneously with inhalation of respirable particulates with potential negative additive effects on health

**Table 2:** Popular types and models of family cars and volumes of their passenger and cargo compartments (http://usnews.rankingsandreviews.com/cars-trucks/Family_Car_Shopping_Space_vs_Fuel_Economy/).

Model	Volume in ft^3^ (m^3^)	
	Passenger compartment	Cargo compartment
*Sedans*		
Hyundai Sonata (4-cyl., manual transmission)	103.8 (2.9)	NA^*∗*^
Kia Optima (4-cyl., manual transmission)	102.2 (2.9)	NA
Honda Accord (4-cyl., automatic transmission)	106 (3.0)	NA
Ford Fusion (4-cyl., automatic transmission)	100.3 (2.8)	NA
*Minivans*		
Honda Odyssey	172.5 (4.9)	38.4 (1.10)
Toyota Sienna (4-cyl.)	164.4 (4.7)	39.1 (1.10)
Kia Sedona	172.3 (4.9)	32.2 (0.91)
Nissan Quest	177.8 (5.0)	25.7 (0.73)
*Compact SUVs*		
Chevrolet Equinox (2WD 4-cyl.)	99.7 (2.8)	31.4 (0.89)
GMC Terrain (2WD 4-cyl.)	99.6 (2.8)	31.6 (0.89)
Hyundai Tucson (2WD, automatic transmission)	101.9 (2.9)	25.7 (0.73)
Mitsubishi Outlander Sport (2WD, automatic transmission)	97.5 (2.8)	21.7 (0.61)
*Midsize SUVs*		
Ford Explorer (FWD)	151.7 (4.3)	21.0 (0.59)
Chevrolet Traverse (FWD)	153.1 (4.3)	24.4 (0.69)
Toyota Highlander (2WD, 4-cyl.)	145.7 (4.1)	10.3 (0.29)
Ford Flex (FWD)	155.8 (4.4)	20.0 (0.57)
GMC Acadia (FWD)	154.0 (4.4)	24.1 (0.68)
Honda Pilot (FWD)	153.7 (4.4)	18.0 (0.51)

Average	134.0 (3.8)	23.97 (0.68)

^*∗*^Not applicable as sedans have a separate trunk or cargo compartment physically separated from the passenger area.

**Table 3:** Types of microbial pathogens and their possible sources in the family car.

Type	Examples	Possible source(s)
Vegetative bacteria	*Legionella pneumophila*; *Pseudomonas aeruginosa; Staphylococcus aureus *(including methicillin-resistant ones)	Biofilms, human occupants, dust, heating/cooling systems, windshield washer fluid, and splashes from road puddles
Mycobacteria	*Mycobacterium tuberculosis; Mycobacterium avium*	Human occupants and biofilms
Bacterial spores	*Bacillus subtilis; B. cereus; Clostridium difficile*	Road dust, upholstery, heating/cooling systems, carpets, human occupants, and pets
Fungi & fungal spores	*Aspergillus niger; Candida albicans*	Road dust, upholstery, heating/cooling systems, carpets, human occupants, and pets
Viruses	Noroviruses; rhinoviruses; influenza viruses; rotaviruses	Human occupants, pets & animal (chickens, pigs) cargo

**Table 4:** Summary of findings on microbes on surfaces in family cars.

Types of surfaces sampled	Steering wheel, radio knob, dashboard, door handle, seat, children's car seat, change holder, window opener, cup holder, seat belt, and area with a food spill
Type of vehicle tested	Higher levels of bacterial contamination in vans and sports utility vehicles than in sedans, possibly due to higher passenger capacity and more frequent transport of children
Variables considered	Different sites inside, type of vehicle, use of the vehicle for transporting children, and geographic location as well as sex and marital status of the drivers
Frequency of occurrence of fungi	Directly related to the mean air temperature of the city where the automobile was located
Frequency of occurrence of bacteria	Directly related to the mean average monthly rainfall as well as air temperature

**Table 5:** Approaches to reducing health risks from pollutants inside family cars.

Approach	Strengths	Limitations
Opening windows for fresh air	Occupant-controlled action with immediate impact on air quality	Noise and increased exposure to road dust & insects
Regular vacuuming and general cleanup of the car interior	A generic means for reducing the accumulation of dust, infectious agents, and allergens on upholstery, carpets, and other surfaces	Such cleaning is often quite infrequent or may be cursory when carried out; it also cannot address the issue of ongoing entry of airborne pollutants from external sources; further, it can reaerosolize settled pathogens for aerial spread/deposition on clean surfaces
Maintenance of air-conditioning & heating systems	Reduction in accumulation of dust as well as build-up of biofilms	Not within the resources or skill sets of most car owners
Prophylactic vaccination	The use of safe & effective vaccines, including those against seasonal influenza, can offer protection	The number of safe and effective vaccines remains limited; certain types of vaccines offer only transient protection and also may not cover “new” pathogens or those with changing antigenic profiles
Installation of a safe and cost-effective air decontamination device	The use of a validated technology may reduce exposure to a variety of airborne pollutants	If such a device is not maintained properly, it could in itself become a sources of airborne pollutants

**Table 6:** Desirable attributes of in-car air decontamination devices.

Attribute	Reason(s) for consideration
Broad-spectrum of activity	Should be able to deal with airborne infectious agents and allergens as well as respirable particulates, odors, and VOCs
Economical to install, maintain, and operate	Must be lightweight not to add significantly to fuel consumption; should indicate when filters & bulbs may require changing
Noise level	Should be as low as possible
Installation or retrofit in all makes of vehicles	Should be capable of ready retrofit
Nontoxic & environmentally friendly	Must be as “green” as possible
